# Review of the Scientific Literature on Young Adults Related to Cardiovascular Disease Intervention

**DOI:** 10.31372/20200501.1084

**Published:** 2020

**Authors:** Dieu-My T. Tran, Angela Sojobi

**Affiliations:** University of Nevada, Las Vegas, Nevada, United States

**Keywords:** young adults, intervention, cardiovascular risk factors, diet, physical activity

## Abstract

Many young adults are at risk for cardiovascular disease related to their behavioral choices. Irresponsible alcohol consumption, tobacco smoking, sedentary lifestyle, poor dietary habits, and excessive weight gain are some of the behaviors that put young adults at risk. The Centers for Disease Control and Prevention identified that 15% of young adults are diagnosed with chronic illnesses related to their behavioral choices. The purpose of this review is to identify, in the literature, interventions that are currently available to young adults and evaluate the adequacy and effectiveness of those interventions. An extensive electronic search was conducted using CINAHL, EBSCOhost, Cochrane, PubMed, and Google Scholar. A total of 130 articles were identified and 28 articles met the inclusion criteria. Three main interventions were identified for young adults: personalized interventions, technology-based interventions, and educational/behavioral interventions. The interventions were all effective to different degrees and interventions were most effective when they were combined. This review impacts in what manner nurses and health care providers deliver health promotion, prevention, and management of cardiovascular risk factors in young adults; in particular, nurses play a key role in lifestyle modifications including diet and exercise.

Cardiovascular disease (CVD) remains the number one cause of death globally and Asian countries contribute significantly to the global burden of CVD (Benjamin, Muntner, & Bittencourt, 2019). As a subpopulation, CVD and cardiovascular risk factors are rapidly increasing in the Asian Indian population, specifically, they have a high prevalence of hypertension and diabetes (Gholap, Davies, Patel, Sattar, & Khunti, 2011; Nag & Ghosh, 2013). A study analyzed the National Health Interview Survey (NHIS) from 2003 to 2005 assessing the prevalence of CVD risk factors among Asian Americans compared to non-Hispanic Whites in the United States found that Asian Indians had higher odds of physical inactivity compared to Whites (2009). Additionally, compared to Whites, Filipinos were more likely to have hypertension and Asian Indians were more likely to have diabetes (Ye, Rust, Baltrus, & Daniels, 2009). Considering that CVD is the number one killer, it affects all races, ethnicities, and ages. However, CVD and modifiable cardiovascular risk factors are preventable; therefore, young adults are the ideal population to implement CVD prevention programs.

Young adults experience a period of identity development where they cultivate a sense of self and acquire autonomy (Benson & Elder Jr, 2011). As such, their transition to adulthood, moving away from the supervision and guidance of their parents or guardians, makes them vulnerable to experimenting and being influenced by peers (Steinberg et al., 2013). Young adults often make decisions and participate in activities that will have long-term health consequences. According to the Centers for Disease Control and Prevention (CDC), two out of three college students drink alcohol excessively or binge drink, and approximately nine out of ten begin smoking between the ages of 18 and 26 (Kann et al., 2018). These behaviors put this age group at a higher risk of developing significant health, social, and psychological problems including CVD, hypertension, liver disease, mental disorders such as depression and anxiety, poor academic performance, family problems, substance use, and risky sexual behaviors (Kann et al., 2018). Additionally, the CDC reports that approximately 1,825 college students die every year from alcohol-related incidents including motor-vehicle accidents.

Furthermore, the majority of young adults, specifically college students, assert their independence by having the option to eat whatever they want instead of what was offered by their parents. The freshman 15 is a phenomenon where students gain more than 15 pounds during their freshman year in college (Levitsky, Halbmaier, & Mrdjenovic, 2004; Mihalopoulos, Auinger, & Klein, 2008). This is attributed to consuming foods high in fat and sugar, high processed foods, and excessive snacking (Mihalopoulos et al., 2008). Studies have found that approximately 37% of college students are obese and many lead a sedentary lifestyle (Burke et al., 2015; Huang et al., 2004; Sarpong, Curry, & Williams, 2017; Tran et al., 2017). The rate of obesity in young adults aged 18–29 years has tripled over the past three decades (National Center for Health Statistics (US), 2009). This is significant considering that obesity during young adulthood increases the likelihood of obesity in older adulthood (age 40 years and older). Also, there is a significant decrease in physical activity level among young adults despite supporting evidence that maintaining the recommended level of activity has significant health benefits (Kann et al., 2018; Kwan, Cairney, Faulkner, & Pullenayegum, 2012; Sabharwal, 2018). Specifically, studies have shown a dramatic decline in physical activity among young adults, especially among those attending college/university (Gordon-Larsen, Adair, Nelson, & Popkin, 2004; Kwan et al., 2012; Zick, Smith, Brown, Fan, & Kowaleski-Jones, 2007).

The CDC reports that 15% of young adults are diagnosed with chronic health conditions related to limited physical activity and poor health choices (Kann et al., 2018). Additionally, for 4–5% of young adults, chronic conditions may further limit their activity levels (US Department of Health and Human Services, 2018). This warrants further evaluation and intervention as these young adults’ lives are being hindered by poor health choices resulting in life-long consequences.

According to the U.S. Census Bureau (2010) young adults constitute 36.5% of the U.S. population; thus, their potential health risks should not be taken lightly. In response to and in understanding the prevalence of cardiovascular health risk behaviors in young adults, it is imperative to identify in the literature the current interventions that are available to young adults and evaluate the adequacy and effectiveness of those interventions. Therefore, the purpose of this review was to examine intervention studies related to young adults’ cardiovascular risk factors.

## Methods

Our literature searches specifically targeted young adults aged 18–39 years and intervention studies targeting cardiovascular risk factors (smoking status, physical inactivity, diet, elevated blood pressure or hypertension, diabetes, hyperlipidemia, overweight/obesity, stress, or alcohol consumption). We conducted an extensive electronic search of the medical literature using CINAHL, EBSCOhost, Cochrane, PubMed, and Google Scholar databases. Keywords included *young adults, college students, cardiovascular risk factors, cardiovascular disease, blood pressure, elevated blood pressure, hypertension, intervention, diabetes, diet, alcohol consumption, binge drinking, physical activity, smoking status, stress, and overweight/obesity*. We limited our search to only include articles of English language and those published between 2000 and 2018. The initial search identified 130 articles. Next, each author (D.M.T. and A.S.) independently reviewed the abstracts and papers, including the reference lists, to determine inclusion criteria. The inclusion criteria included a form of intervention implementation on at least one or more cardiovascular risk factors in young adults or college students. A total of 28 studies met the inclusion criteria for review.

## Results

### Study Details

The majority (92%) of the studies included in this review were conducted in the United States. The articles were comprised of 23 randomized control trials (RCTs), one quasi-experimental study, one longitudinal study, and four systematic reviews (Table 1). Sample sizes varied among the studies ranging from 49 to 1,639 young adults. Among the 28 articles, nine were intervention studies focused on reducing alcohol consumption, thirteen on promoting weight management, five promoting healthy eating, seven on physical activity and healthy lifestyle, and one focused on reducing elevated blood pressure and stress levels. The following intervention themes emerged and were identified and categorized: personalized interventions, technology-based interventions, and educational/behavioral interventions.

**Table 1:** Reviewed Articles Description (n = 27)

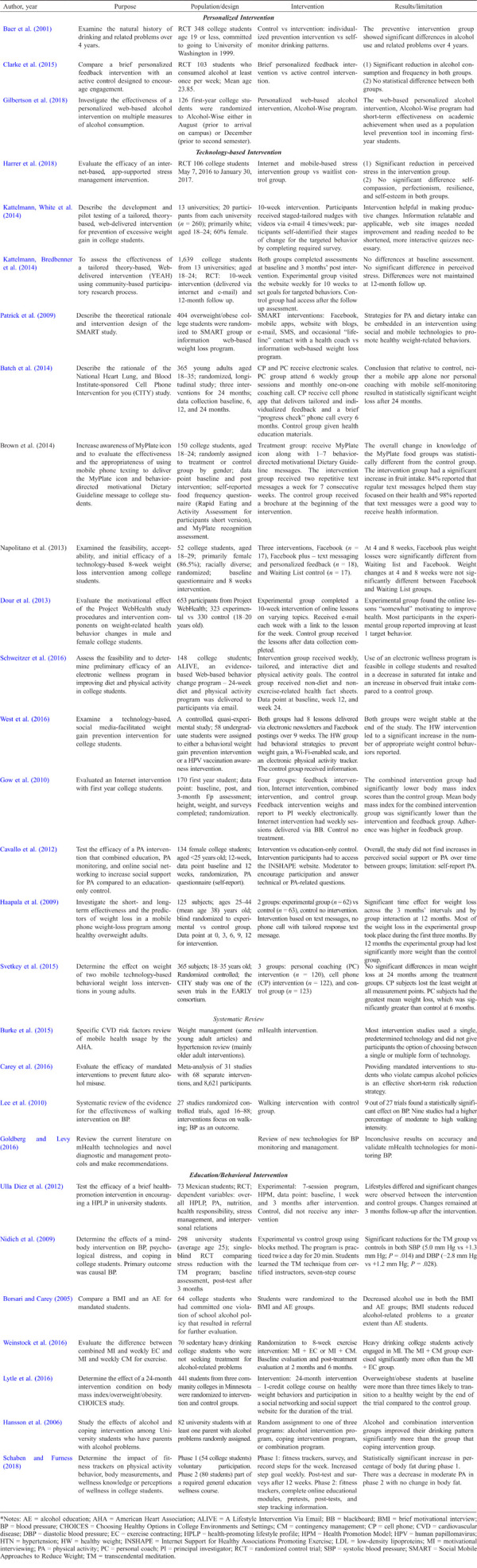

#### Personalized Intervention

Among the 28 reviewed articles, three evaluated the effectiveness of personal interventions to reduce alcohol consumption in college students. Baer, Kivlahan, Blume, McKnight, and Marlatt (2001) conducted a RCT to compare the effect of individualized prevention interventions to self-monitor drinking patterns in 348 high-risk college student drinkers over a four-year period. Clarke, Field, and Rose (2015) studied the effect of a brief personalized feedback intervention to an active control group in 103 high-risk college students who consumed alcohol at least once a week. Baer et al. (2001) found statistically significant effects of personalized interventions in their intervention groups compared to the control groups. However, Clarke et al. (2015) found no significant differences between the personalized intervention and active control groups.

Gilbertson, Norton, Beery, and Lee (2018) implemented a personalized RCT intervention, Alcohol-Wise, on college students (an intervention group and a waitlist group) who were alcohol drinkers (*n* = 126) using web-based technology. The intervention group was administered the Alcohol-Wise intervention in their first semester in August (prior to arrival on campus) and the waitlist group received the intervention second semester in December (prior to starting the second semester). The study found that there was a higher GPA in the intervention group (first semester) whereas there was an initial increase in alcohol consumption in the waitlist group (second semester) at 10 weeks. At 24 weeks, academic achievement was higher in the intervention group compared to the waitlist group; however, the intervention group also demonstrated an increase in frequency of heavy episodic drinking compared to the waitlist group.

#### Technology-Based Intervention

Among the articles reviewed, 14 evaluated the effectiveness of technology-based interventions on the following topics: improving diet and physical activity, preventing weight gain, stress management, and increasing social support. Sample sizes ranged from 106 to 1,639 participants. Technology-based interventions were mainly delivered via mobile phones using text messaging, phone applications, and internet through email and social media. Harrer et al. (2018) compared a stress management intervention that was delivered through the internet and mobile devices to a control group. The intervention group had a significant reduction in perceived stress compared to the control group. A Youth Experience and Health (YEAH) study also examined the effect of web-delivered interventions on stress and found no significant difference in perceived stress between the intervention and control groups (Kattelmann, White et al., 2014). Likewise, when education and physical activity were compared to online social networking to improve social support for activity, the researchers found no differences in perceived social support or physical activity in either group (Cavallo et al., 2012). However, a 10-week intervention whereby participants received video reminders through email 4 times a week resulted in participants making productive behavioral changes (Kattelmann, Bredbenner et al., 2014). The majority of the reviewed articles in this category were RCT studies that delivered the interventions electronically using social networking to impact weight management and healthy food choices. Six of the studies found that the intervention group had significant weight loss and made healthier food choices compared to the control group (Brown, O’Connor, & Savaiano, 2014; Haapala, Barengo, Biggs, Surakka, & Manninen, 2009; Gow, Trace, & Mazzeo, 2010; Napolitano, Hayes, Bennett, Ives, & Foster 2013; Patrick et al., 2009; West et al., 2016). Five of the studies found no significant differences between the intervention and control groups (Batch et al., 2014; Dour et al., 2013; Kattelmann, Bredbenner et al., 2014; Schweitzer, Ross, Klein, Lei, & Mackey, 2016; Svetkey et al., 2015).

Also included in this review were systematic reviews on studies that uses technology-based interventions. For instance, Carey, Scott-Sheldon, Garey, Elliott, and Carey (2016) reviewed 31 studies, which included 68 interventions to prevent alcohol misuse in college students and involve 8,621 participants. They found that having students who violate campus alcohol policies participate in mandated interventions only had a short-term effect in reducing risk of alcohol misuse. Likewise, Lee, Watson, Mulvaney, Tsai, and Lo (2010) reviewed 27 RCT studies on the effect of walking on blood pressure, one third of the studies found a statistically significant effect of walking on lowering blood pressure. A review of current literature on the effectiveness of digital health innovations (cuffless blood pressure sensors, wireless smartphone-enabled upper arm blood pressure monitors, mobile applications, and remote monitoring technologies) on hypertension control demonstrated that digital health innovations improve hypertension and medication adherence (Goldberg & Levy, 2016). Burke et al. (2015) reviewed studies conducted between 2004 and 2014 on the use of technology mediated tools to improve health. They concluded that technology mediated tools are impactful in delivering health related messages efficiently, sharing of self-management parameters between patients and clinicians, and delivering of feedback and guidance to patients.

#### Educational/Behavioral intervention

Among the reviewed articles, seven studied the effect of educational and behavioral interventions on health-promoting lifestyles, elevated blood pressure, increased physical activity, weight management, and alcohol consumption. Ulla Diez et al. (2012) conducted an RCT (*n* = 73) to test the efficacy of the Health Promotion Model to encourage a health promoting lifestyle in two groups, an intervention and control group. There was a significant lifestyle change in the intervention group that was also sustained at the 3-month follow up compared to the control group. Likewise, two RCT studies (*n* = 134 and 298) compared the impact and feasibility of educational interventions, mind/body interventions, and behavioral interventions to reduce blood pressure and increase exercise in students with elevated blood pressure and obesity. Results identified significant differences among the study outcome variables (reduced systolic and diastolic blood pressure and increased exercise) in the intervention group compared to the control group (Nidich et al., 2009; Schaben & Furness, 2018).

Two research groups conducted interventions on alcohol consumption using an educational session (Borsari & Carey, 2005; Hansson, Rundberg, Zetterlind, Johnsson, & Berglund, 2006). Borsari and Carey (2005; *n* = 64) compared the effect of a brief motivational interview and an alcohol educational session in college students who drank alcohol. While there was decreased alcohol consumption in both groups, the brief motivational interview group demonstrated a greater reduction in alcohol related problems. Likewise, Hansson and colleagues (2006; *n* = 82) studied the effect of an alcohol intervention, coping intervention, and a combination of both the alcohol and coping interventions and found that the alcohol intervention and the combined intervention were both more effective in reducing alcohol consumption compared to the coping intervention alone.

Two research groups studied motivational and conditioning interventions (Lytle et al., 2016; Weinstock, Petry, Pescatello, & Henderson, 2016). Weinstock and colleagues (2016; *n* = 70) compared the effect of combined motivational interviewing and weekly exercise to the combined motivational interviewing and weekly contingency management for exercise on alcohol consumption. They found that the combined intervention groups exercised significantly more and had a decrease in binge drinking episodes. However, the effect was more significant when they used a combination of motivational interviews and the weekly contingency management for the exercise group. Lytle et al. (2016; *n* = 441) evaluated the effect of a 24-month intervention on weight management compared to a control group. They found that the intervention group was three times more likely to transition to a healthy weight compared to the control group. These studies have shown that behavioral interventions are effective on increasing activity and controlling weight gain, blood pressure, and alcohol consumption in college students. More importantly, the effect is heightened when behavioral interventions are combined with education interventions.

## Discussion

The literature search yielded 130 articles with 26 studies meeting the inclusion criteria for this review. In their transition from adolescence to adulthood, young adults may feel invincible and carry out behaviors that could have long-term health sequelae (Benson & Elder Jr, 2011). They may adopt lifestyle habits such as binge drinking, smoking, making unhealthy food choices, and becoming sedentary that place them at increased risk for CVD. Several studies examined the effectiveness of different interventions in young adults with cardiovascular risk factors to prevent their risk for CVD in the future. Though there are many intervention choices, they are not equally effective to mitigate risk factors in young adults.

The studies we evaluated that used personalized interventions demonstrate effectiveness for reducing alcohol consumption, weight loss, and blood pressure control. However, the effect was not equally effective throughout each semester for college students. Nor was the effect on weight and blood pressure sustained. Based on the studies reviewed, it is worthwhile for health care providers to keep in mind that there were not enough studies completed in young adults using personalized interventions to make a strong conclusion for recommendations.

Considering the global evolution of technology and social media, it was not surprising to discover that technology-based interventions were effective in promoting healthy behaviors and behavior modifications that improve healthy food choices, physical activity, weight management, and stress reduction. Though technology-based interventions were effective, the outcomes were short lived compared to outcomes of behavioral and combined interventions. Behavioral interventions seem to demonstrate an impact on health-promoting lifestyle changes (Borsari & Carey, 2005; Lytle et al., 2016). Once again when reviewing the technology-based interventions, the most effective interventions were a combination of interventions instead of a single intervention (Gow et al., 2010; Hansson et al., 2006; Weinstock et al., 2016). It is worth noting that the advancement in technology also plays a factor in the type of interventions that were offered which may explain why there are more technology-based interventions available in the literature compared to the other two types of interventions.

Notably, some of the intervention studies were implemented over a short period while others were over a longer period, ranging from 10 to 24 months. It is unclear whether the duration of the interventions influenced the effectiveness of the intervention. Therefore, it would be beneficial to determine in future studies whether there is a significant difference in the duration of certain interventions on their effectiveness.

## Conclusions and Recommendations for Future Research

There are several factors that could impact the effectiveness of interventions. In this review, duration of the interventions varied significantly. As such, future longitudinal studies to determine if the duration of the intervention influenced effectiveness of the interventions would be beneficial. Additionally, obtaining a sustainable effect of the studied health outcomes is as important to achieve and warrants further investigation. As well, the majority of the reviewed articles were RCTs; therefore, it worthwhile to consider.

Poor eating habits, lack of exercise, excessive alcohol consumption, and excessive weight gain are some of the factors that endanger the health of young adults including young Asian Americans who are fast-growing in the United States with high prevalence of CVD that impact the current and future public health. According to the CDC and the National Institute of Alcohol Abuse and Alcoholism statistics (2018), a significant number of college students and young adults die yearly from alcohol related-incidents as well as being involved in alcohol-related physical and sexual assaults. Likewise, many college students and young adults are at risk for chronic illnesses related to their health risk behaviors. This review identified, analyzed, and synthesized published literature on interventions to decrease CVD risk factors in young adults, which can greatly contribute to the literature including guiding clinicians and researchers to identify the most appropriate and effective interventions to help young adults lead and maintain a healthy lifestyle. Additionally, this review impacts in what manner nurses and health care providers deliver health promotion, prevention, and management of cardiovascular risk factors in young adults; in particular, nurses play a key role in lifestyle modifications including diet and exercise.

## Acknowledgments

None.

## Declaration of Conflicting Interests

The authors declared no potential conflicts of interest with respect to the research, authorship, and/or publication of this article.

## Funding

None.
